# Synthesis of Low Temperature Resistant Hydrogenated Nitrile Rubber Based on Esterification Reaction

**DOI:** 10.3390/polym13234096

**Published:** 2021-11-24

**Authors:** Lin Wang, Yanqiang Ni, Xin Qi, Liqun Zhang, Dongmei Yue

**Affiliations:** 1 State Key Laboratory of Organic-Inorganic Composites, Beijing University of Chemical Technology, Beijing 100029, China; WangL18882029658@163.com (L.W.); niyanqiang@163.com (Y.N.); qixin0930@126.com (X.Q.); zhanglq@mail.buct.edu.cn (L.Z.); 2 Key Laboratory of Beijing City on Preparation and Processing of Novel Polymer Materials, Beijing University of Chemical Technology, Beijing 100029, China

**Keywords:** hydrogenated nitrile rubber (HNBR), grafting, low temperature resistance

## Abstract

Hydrogenated Nitrile Rubber (HNBR) is widely used in aerospace, petroleum exploration and other fields because of its excellent performances. However, there remains a challenge of balancing the oil resistance and the low temperature resistance for HNBR. In this work, a series of grafted carboxyl nitrile rubber (XNBR) was prepared by the esterification reaction between active functional groups (–COOH) of XNBR and alkanols of different molecular chain lengths (C_8_H_17_OH, C_12_H_25_OH, C_16_H_33_OH, C_18_H_37_OH) or Methoxypolyethylene glycols (MPEG) of different molecular weights (Mn = 350, 750, 1000). The structure and low temperature resistance of as-obtained grafted polymers were characterized by Fourier Transform Infrared (FTIR), ^1^H-NMR and Differential scanning calorimetry (DSC). It was found that the glass transition temperatures (T_g_) of grafted XNBR were significantly decreased. MPEG grafted polymers with better low temperature resistance were then selected for hydrogenation. As-prepared hydrogenated XNBR grafted with MPEG-1000 (HXNBR-g-1000) showed the lowest T_g_ of −29.8 °C and the best low temperature resistance. This work provides a novel and simple preparation method for low temperature resistant HNBR, which might be used potentially in extremely cold environments.

## 1. Introduction

Nitrile butadiene rubber (NBR) is a random copolymer of butadiene and acrylonitrile [1] with excellent oil resistance and good mechanical strength, which has been widely used in oil fields, automobiles, wire and cable, adhesives and other fields [2,3]. However, due to the existence of unsaturated bonds on the molecular chain, it shows poor aging resistance. Hydrogenated nitrile rubber (HNBR) is the product of selective hydrogenation of NBR, which retains the original excellent performances of NBR and improves the ability of heat resistance, acid and alkali resistance and aging resistance [4,5,6,7]. However, HNBR is easy to crystallize due to the nearly ordered polyethylene structure of main chain [8,9], which causes an increase of T_g_. As a result, HNBR has poor low temperature resistance. 

In order to improve the low temperature resistance of HNBR, physical modification and chemical modification [10,11] can be adopted. Physical modification includes adding small molecular plasticizer, adjusting vulcanization formula or rubber sharing. Kim D et al. [12] used various amounts and types of plasticizers to investigate the low temperature resistance of NBR materials. They found that T_g_ of NBR decreased when the plasticizer was added. In particular, the addition of Dioctyl adipate (DOA) led to a lower T_g_ than other plasticizers, which decreased T_g_ from −43 to −52 °C. Although the introduction of plasticizers can reduce T_g_, the plasticizer is easy to migrate and exude, and excessive addition will result in deterioration of mechanical and dielectric properties [13,14]. Anna Laskowska et al. [15] investigated the effect of imidazole-based ionic liquids on the properties of NBR composites. Fifteen phr of hydrophobic 1-ethyl-3-methylimidazolium bis(trifluoromethylsulfonyl)imide (EMIM TFSI) ionic liquid decreased T_g_ from −23 to −31 °C. However, the mechanical properties of rubber were greatly affected. Chemical modification mainly involves the introduction of a flexible third monomer into the molecular chain by grafting or copolymerization. In our group [16], epoxidation and esterification reactions were used to introduce a flexible third monomer to HNBR molecular chain, as-obtained HNBR showed non crystallized structure, decreased T_g_ of −34.1 °C and the better low temperature performances. It indicates that the addition of the third monomer can not only reduce T_g_, but also inhibit crystallization.

Carboxyl nitrile butadiene rubber (XNBR) is a special type of nitrile rubber containing carboxyl groups prepared by emulsion copolymerization of butadiene, acrylonitrile and a small amount of unsaturated acids [17]. It has good mechanical performances and resistance with abrasion and oil [18,19,20]. In addition, several kinds of active functional groups of XNBR, such as nitrile (–CN), carboxylic (–COOH) and alkene groups (–C=C) [17,21,22], can participate in reactions to form different types of chemical bonds. For example, the carboxylic functional group can react with several materials, such as metal oxides, amines, polyols and epoxies, which are widely used for the preparation of nano-composites and new materials [23,24].

**Figure 1:**
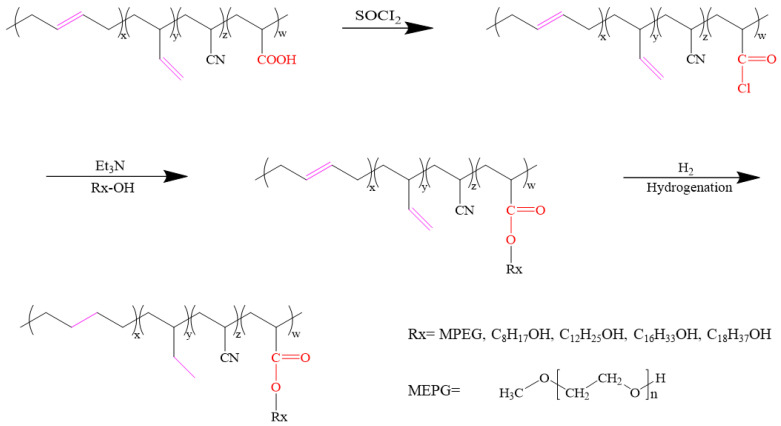
Schematic of XNBR grafting and hydrogenation reactions.

In this work, the flexible monomer with hydroxyl group were grafted to XNBR by the reaction between –COOH and −OH. Subsequently, these grafted XNBR were hydrogenated to fabricate a series of grafted HXNBR (Figure 1). Among them, HXNBR-g-1000 showed the lowest T_g_ of −29.8 °C and the best low temperature resistance. The reaction between functional groups ensures grafting efficiency and avoids the generation of by-products and gels. This work provides a novel and simple method to improve the low temperature resistance for HNBR while maintaining its oil and aging resistance. 

## 2. Materials & Methods

### 2.1. Materials

Carboxyl nitrile rubber with acrylonitrile content of 28%, carboxyl content of 8.5%, and the molecular of 1.5 × 10^4^ was obtained from Zeon Corporation; anhydrous ethanol and chlorobenzene were purchased from Beijing Chemical Plant (Beijing, China); Octanol (C_8_H_17_OH), Dodecanol (C_12_H_25_OH), Cetyl alcohol (C_16_H_33_OH), Stearyl alcohol (C_18_H_37_OH) and Methoxypolyethylene glycols (Mn = 350, 750, 1000) were purchased from Aladdin Reagent Co., Ltd. (Shanghai, China) Triethylamine (Et_3_N) and Thionyl Chloride (SOCl_2_) were purchased from Shanghai Sigma-Aldrich Co., Ltd. (Shanghai, China) Deuterated chloroform was obtained from National Chemical Reagent Co (Beijing, China).

### 2.2. Preparation of Grafted XNBR with Different Side Groups

Ten grams of XNBR was dissolved in 250 mL chlorobenzene, and 10 mL thionyl chloride was then added to the polymer solution dropwise. The reaction was carried out at 78 °C for 4 h. After that, an appropriate amount of C_8_H_17_OH, C_12_H_25_OH, C_16_H_33_OH, C_18_H_37_OH or MPEG (Mn = 350/750/1000) (the molar ratio of −COOH: −OH = 1:1.2) and triethylamine (the molar ratio of −COOH:Et_3_N =1:1.2) were added to the above solution, and the reaction was run at 55 °C for 5 h. The whole reaction process was carried out in nitrogen atmosphere. The products were coagulated in ethanol, washed three times, and dried at 55 °C. As-obtained grafted polymers are called XNBR-g-8, XNBR-g-12, XNBR-g-16, XNBR-g-18, XNBR-g-350, XNBR-g-750, and XNBR-g-1000, respectively.

### 2.3. Hydrogenation of Grafted XNBR with Different Side Groups

Eighteen grams of grafted XNBR with MPEG (Mn = 350/750/1000) and 282 mL of chlorobenzene were added into an autoclave. Then, the required catalyst was transferred into the reactor. The autoclave was slowly heated and maintained at 120 °C for 8 h. The whole reaction process was carried out in hydrogen atmosphere. The products were coagulated in ethanol, washed thoroughly and dried at 55 °C. As-prepared products are marked as HXNBR-g-350, HXNBR-g-750, HXNBR-g-1000, respectively.

### 2.4. Characterization

Fourier Transform Infrared (FTIR) spectra were obtained on a Bruker Tensor 27 spectrophotometer (Bruker, Karlsruhe, Germany) in the range of 400 to 4000 cm^−1^ with a resolution of 4 cm^−1^, with Attenuated Total Reflectance (ATR) mode. Nuclear Magnetic Resonance (NMR) measurements were carried out on an AV600-400MHZ spectrophotometer (Bruker, Karlsruhe, Germany ) using CDCl_3_ and C_3_D_6_O solution as solvents. The element content was determined using the CHNSO element analyzer produced by Elementor, Hanau, Germany. Differential scanning calorimetry (DSC) was performed on Mettler Toledo (Zurich, Switzerland ). Firstly, the sample was heated from 25 to 80 °C at a rate of 10 °C/min and kept at 80 °C for 5 min. Then, it was cooled from 80 to −100 °C at a rate of 10 °C/min and kept at −100 °C for 5 min under nitrogen atmosphere. Ultimately, the sample was heated from −100 to 100 °C at a rate of 10 °C /min. The glass transition temperature was determined based on the heating curve from −100 to 100 °C at a rate of 10 °C /min.

Thermogravimetric analysis (TGA) measurements were performed on Mettler Toledo equipment from 25 to 600 °C at a heating rate of 10 °C /min under nitrogen atmosphere.

## 3. Results and Discussion

### 3.1. Structures of Grafted XNBR

**Figure 2:**
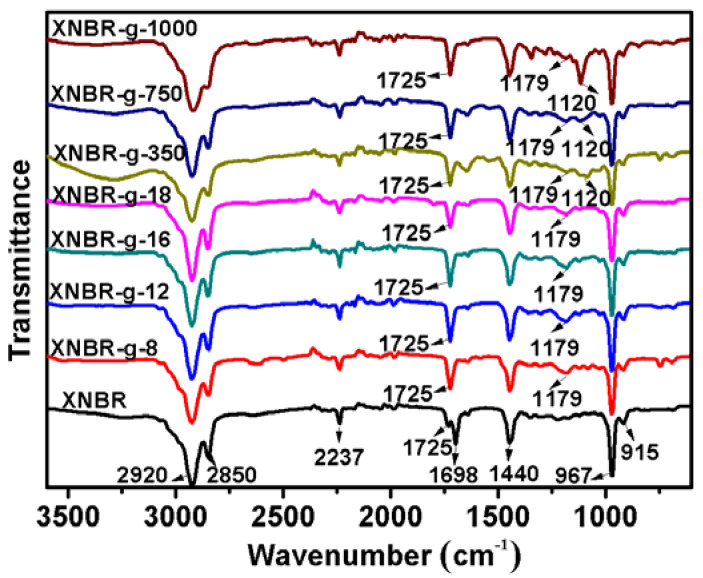
FTIR spectra of XNBR and XNBR grafted with different side groups.

A series of alkanols (C_8_H_17_OH, C_12_H_25_OH, C_16_H_33_OH, C_18_H_37_OH) and Methoxypolyethylene glycols (Mn = 350, 750, 1000) were grafted to XNBR, and the structures of the grafted polymers were characterized using FTIR and ^1^H-NMR. The FTIR spectra of XNBR and grafted XNBR are shown in Figure 2. The absorption peaks at 2920 and 2850 cm^−1^ are attributed to the stretching vibration of –CH_2_. The bands at 967 and 915 cm^−1^ are assigned to the bending vibration of =C–H of the butadiene units. The peak at 2237 cm^−1^ is attributed to cyano group [25,26]. The band at 1725 cm^−1^ is due to the stretching vibration of C=O [27]. In the case of XNBR, the weak peak at 1725 cm^−1^, attributed to C=O, and the strong peak at 1698 cm^−1^, ascribed to the migration absorption peak of hydrogen bond in the carboxyl group, are observed. After grafting, the peak at 1698 cm^−1^ disappears completely and the peak intensities at 1725 cm^−1^ increase. In addition, the peaks at 1179 and 1120 cm^−1^ are attributed to the stretching vibration of –COOC– and –C–O–C–, respectively [28]. It indicates that the grafting reaction was successful and ester groups were formed by the grafted side chains.

**Figure 3:**
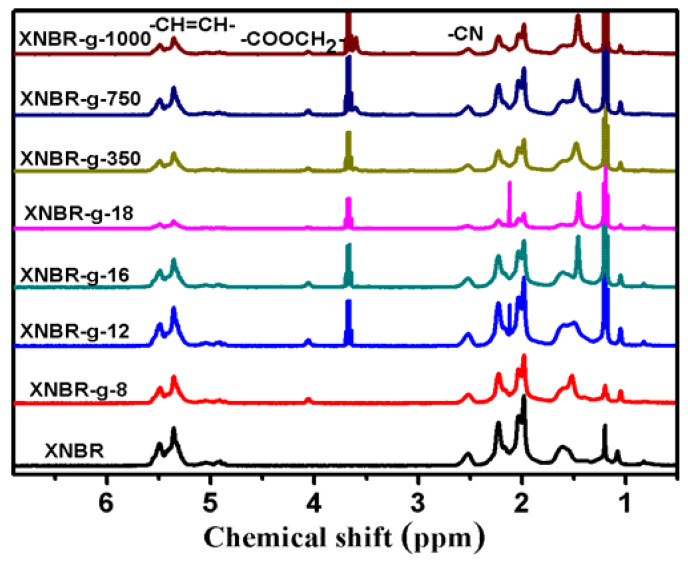
^1^H-NMR spectra of XNBR and XNBR grafted with different side groups.

The ^1^H-NMR spectra of XNBR and grafted XNBR are shown in Figure 3. The peaks in the range of 4.8–5.8 ppm are attributed to the protons of −HC=CH−. The peaks of 1.0–2.5 ppm are assigned to the saturation protons. The peak at 2.53 ppm indicates the proton of –CN [5,25]. The successful grafting reactions are confirmed by the presence of the new peak at 4.2 ppm, which is ascribed to the proton of –COOCH_2_− [29,30].

In order to further confirm the success of grafted reactions, elemental analysis was performed for XNBR grafted with different side groups. As shown in Table 1, XNBR has the highest carbon and nitrogen content as well as the lowest oxygen content. The content of nitrogen for the grafted polymers decreases from 6.67% to 6.23% when the molecular weight of the grafted side groups increases. It indicates that the grafting reaction was successful. The nitrogen element only comes from the nitrogen atom of the cyano group, and there is no nitrogen element in the grafted side groups. As the molecular weight of the grafted side group increases, the nitrogen element in the grafted polymer decreases correspondingly. 

**Figure 4:**
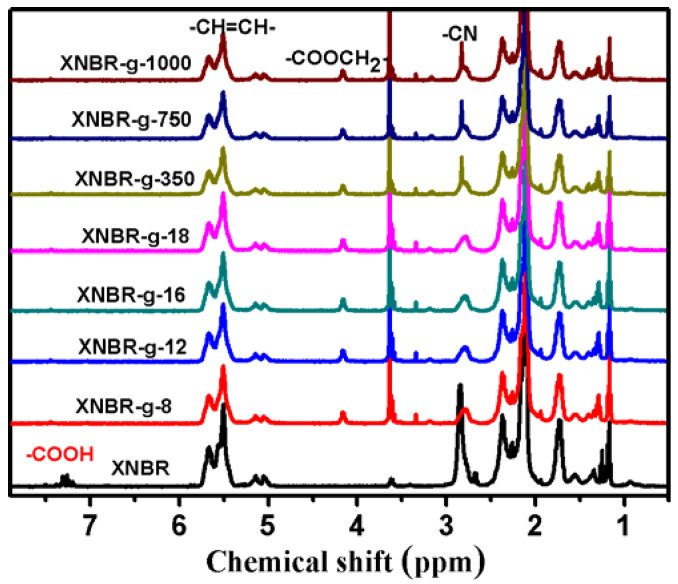
^1^H-NMR spectra of XNBR and XNBR grafted with different side groups using C_3_D_6_O.

In order to characterize the esterification rate of nitrile rubber with different alkanols and methoxypolyethylene glycols, deuterated acetone was selected as the nuclear magnetic reagent to compare the content of residual carboxyl groups before and after the grafting reaction. As shown in Figure 4, XNBR shows a carboxyl proton absorption peak at 7.3 ppm before grafting. However, the peak at 7.3 ppm disappears after grafting, indicating that the esterification reaction was complete.

**Figure 5:**
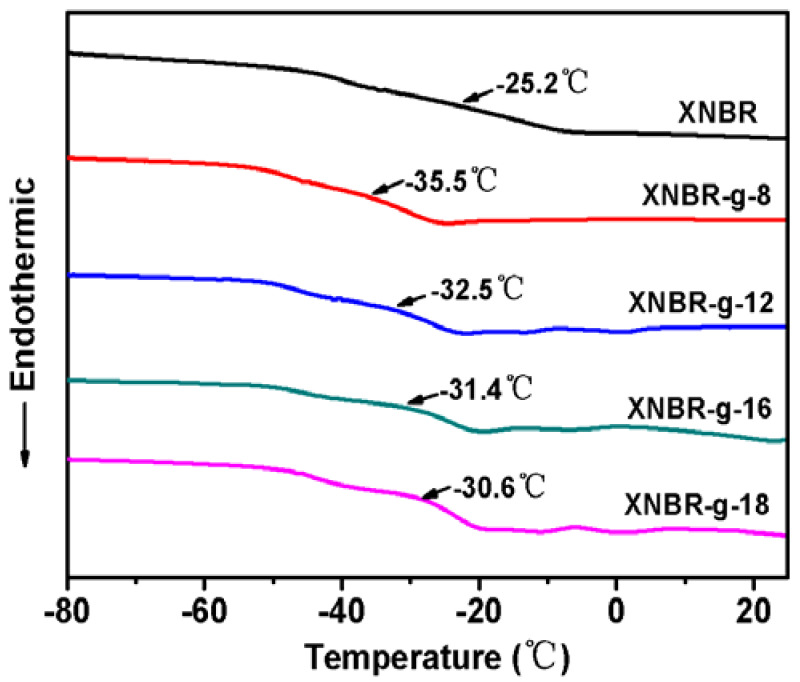
DSC curves of XNBR and XNBR grafted with different long-chain alkanols.

Glass transition temperature (T_g_) is a significant physical parameter that has a great influence on the application performance of rubber. Figure 5 shows DSC curves of XNBR and XNBR grafted with different long-chain alkanols. XNBR shows a T_g_ of −25.2 °C. T_g_ of all grafted XNBR are much lower than that of XNBR. As the carbon atoms of alkanol increases from 8, 12, 16 to 18, the T_g_ of the grafted XNBR gradually increases from −35.5, −32.5, −31.4 to −30.6 °C. When the length of the molecular chain for alkanol grows, T_g_ of grafted XNBR gradually increases. As the length of the alkanol chain increases, the steric hindrance effect increases, which hinders the internal rotation of the molecular chain, resulting in a decrease of segment flexibility and an increase of T_g_. 

**Figure 6:**
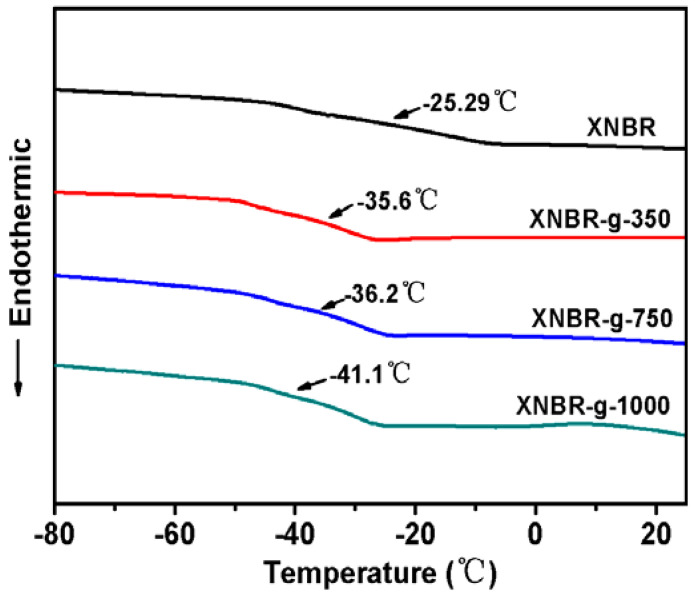
DSC curves of XNBR and XNBR grafted with MPEG of different molecular weights.

Figure 6 shows DSC curves of XNBR and XNBR grafted with MPEG of different molecular weights. Compared to the pristine XNBR, the grafted XNBR show lower T_g_. As the molecular weight of MPEG increases from 350, 750 to 1000, T_g_ of the grafted XNBR gradually decreases from −35.6, −36.2 to −41.1 °C. MPEG contains flexible ether groups. As the flexible side groups increase, the distance between polymer molecules increases and the interaction weakens. In another words, the “internal plasticization” is produced, hence T_g_ of grafted XNBR eventually decreases. Therefore, we choose MPEG grafted polymer with better low temperature resistance for hydrogenation.

### 3.2. Structures of Grafted HXNBR

**Figure 7:**
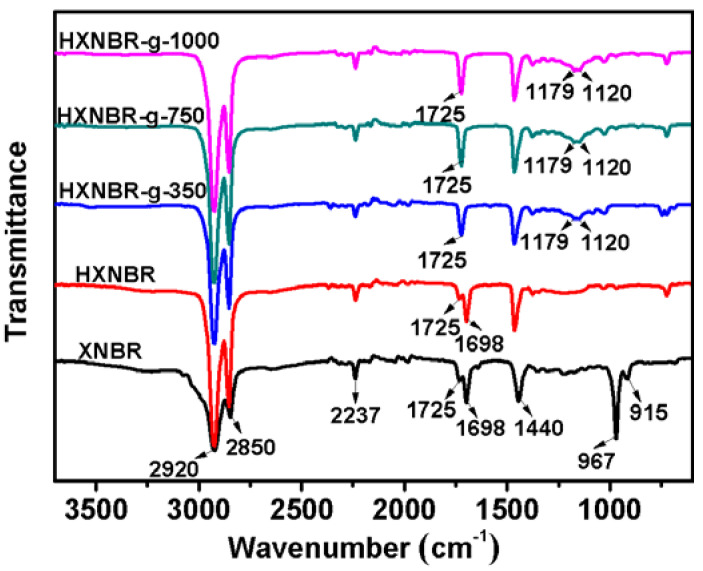
FTIR spectra of XNBR, HXNBR and HXNBR grafted with MPEG of different molecular weights.

**Figure 8:**
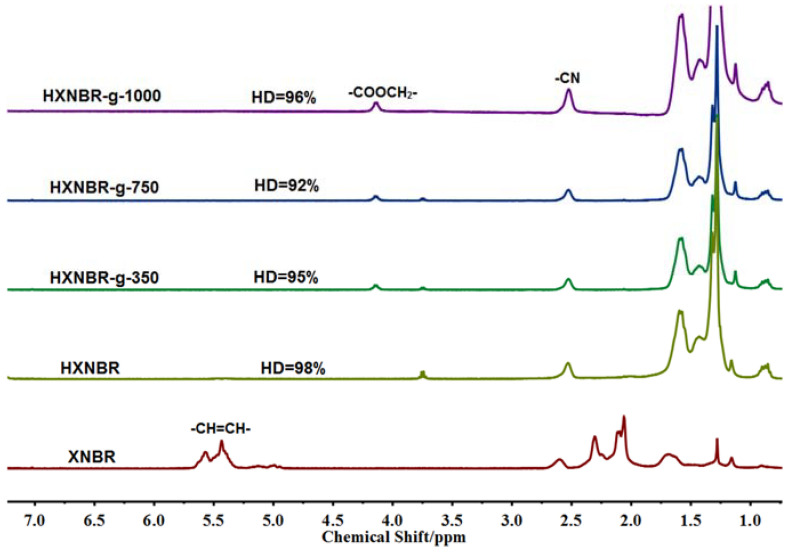
^1^HNMR spectra of XNBR, HXNBR and HXNBR grafted with MPEG of different molecular weights.

XNBR grafted with different molecular weights of MPEG were selected for hydrogenation, and the structure of the hydrogenated products were characterized by FTIR and ^1^H-NMR (Figure 7 and Figure 8). As can be seen from IR spectra in Figure 7, the absorption peaks at 967 and 915 cm^−1^ of XNBR indicate the presence of unsaturated C=C in the pristine rubber. After hydrogenation, HXNBR and HXNBR grafted with different MPEG side groups have no obvious absorption peaks at 967 and 915 cm^−1^, indicating that the hydrogenation reaction for the C=C bond is successful.

As shown in Figure 8, XNBR shows the proton absorption peaks of –CH=CH− in the range of 4.8–5.8 ppm. However, the hydrogenated grafted products have no absorption peaks in this range, indicating that the C=C bond is saturated to the C–C bond. According to the integral area of nuclear magnetic peaks, the hydrogenation degree of the hydrogenated grafted products is above 90%.

**Figure 9:**
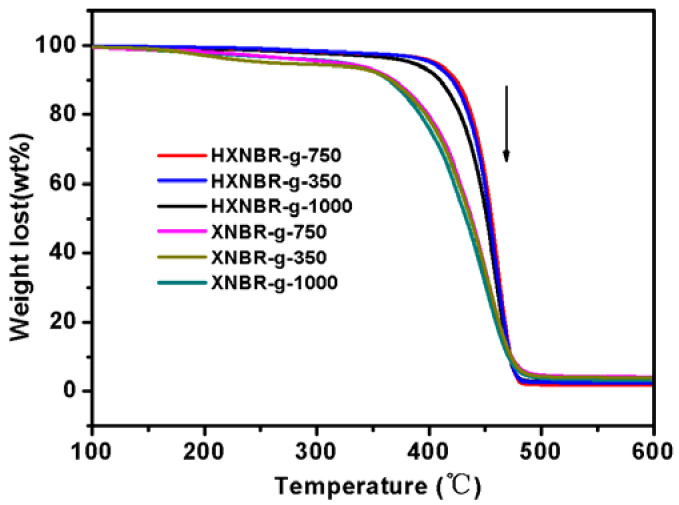
TG curves of HXNBR and XNBR grafted with MPEG of different molecular weights.

The thermogravimetric test was used to analyze the thermal properties of XNBR and HXNBR grafted with MPEG of different molecular weights. TG curves of grafted and hydrogenated products is shown in Figure 9, respectively. Grafted HXNBR start to decompose at above 380 °C, while the initial decomposition temperatures for grafted XNBR are in the range of 150–180 °C. All hydrogenated products show higher thermostabilities than grafted XNBR before hydrogenation. The thermal decomposition temperature is less affected by different side groups. It can be found that the thermal stability of hydrogenated products is improved obviously.

**Figure 10:**
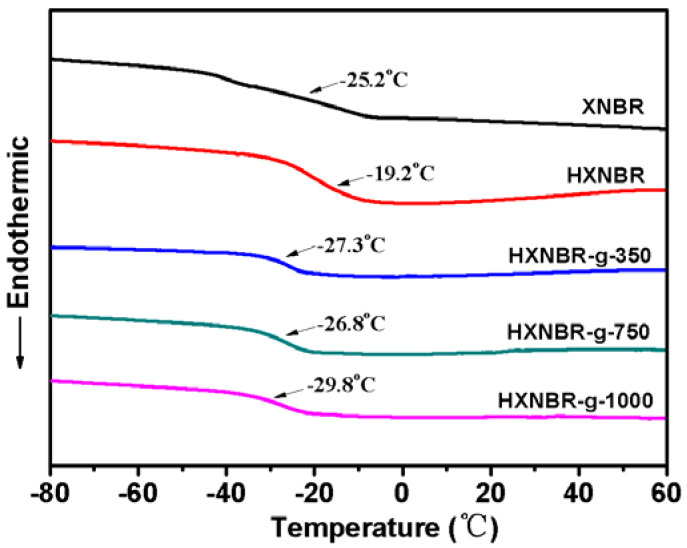
DSC curves of HXNBR and HXNBR grafted with MPEG of different molecular weights.

DSC curves of HXNBR and HXNBR grafted with MPEG of different molecular weights are shown in Figure 10. Compared to T_g_ of −25.2 °C for the pristine XNBR, HXNBR shows a higher T_g_ of −19.2 °C. Due to the decrease of unsaturated bonds on the main chain after hydrogenation, the flexibility of the molecular chain segment decreases. However, T_g_ of HXNBR grafted with different MPEG side groups ranges from −26.8 to −29.8 °C. It can be seen that the low temperature resistance of the grafted HXNBR has been greatly improved. Among them, HXNBR-g-1000 with the lowest T_g_ of −29.8 °C has the best low temperature resistance. The side group of HXNBR-g-1000 is MPEG with the largest molecular weight of 1000, which contains the most flexible ether bond. Therefore, the molecular chain segment of HXNBR-g-1000 with more flexible side groups exhibits the best flexibility.

HXNBR-g-1000 with the best low temperature resistance was compared with H2865C, an imported HNBR with similar acrylonitrile content. H2865C was prepared by hydrogenation of lanxess 2865C NBR. As shown in Table 2, HXNBR-g-1000 has a lower T_g_ and better low temperature resistance than H2865C.

## 4. Conclusions

A series of grafted XNBR was prepared using an esterification reaction between –COOH of XNBR and -OH of flexible monomer. By grafting different molecular weights of MPEG, T_g_ of the grafted polymer is significantly decreased, and XNBR-g-1000 shows the lowest T_g_ of −41.1 °C. Subsequently, the hydrogenation of XNBR grafted with MPEG was carried out. Among them, HXNBR-g-1000 shows the best low temperature resistance, with a T_g_ of −29.8 °C, which is reduced by 10.6 °C compared with the original HXNBR. This work provides a novel and simple preparation method for low temperature resistant HNBR, which might be used potentially in extremely cold environments.

## Figures and Tables

**Table 1:** Elemental analysis of XNBR and XNBR grafted with different side groups.

Samples	C [%]	N [%]	O [%]	H [%]
XNBR	79.84	6.68	4.10	9.38
XNBR-g-8	76.83	6.67	6.85	9.65
XNBR-g-12	77.75	6.61	5.93	9.71
XNBR-g-16	79.45	6.54	4.35	9.66
XNBR-g-18	79.06	6.49	4.76	9.69
XNBR-g-350	78.15	6.47	5.73	9.65
XNBR-g-750	75.01	6.36	9.19	9.44
XNBR-g-1000	72.13	6.23	12.36	9.28

**Table 2:** Comparison of low temperature resistance of different HNBR.

Brand	Acrylonitrile (wt%)	HD (%)	T_g_ (°C)
HXNBR-g-1000	28	96	−29.8
H2865C	28	98	−23.2

## Data Availability

The data presented in this study are available on request from the corresponding author.
